# Interaction of β-Caryophyllene with a Simplified Membrane Model and Its Growth-Inhibitory Effect Against *Escherichia coli* ATCC 25922

**DOI:** 10.3390/pathogens15090887

**Published:** 2026-08-24

**Authors:** Noé Luiz-Santos, Juan Luis Morales-Landa, Jesús Carlos Ruiz-Suárez, Estefania Lazcano-Díaz

**Affiliations:** 1 Centro de Investigación y Asistencia en Tecnología y Diseño del Estado de Jalisco A.C. Subsede Noreste, vía de la Innovación 404, Parque PIIT, Apodaca 66628, Nuevo León, Mexico; 2 Centro de Investigación y de Estudios Avanzados del Instituto Politécnico Nacional, vía del Conocimiento 201, Parque PIIT, Apodaca 66600, Nuevo León, Mexico

**Keywords:** natural antimicrobial compounds, essential oils (EOs), membrane biophysical analysis, differential scanning calorimetry (DSC)

## Abstract

The increasing prevalence of antimicrobial resistance in bacteria highlights the need for alternative membrane-active compounds with favorable safety profiles. β-Caryophyllene (BCP), a bicyclic sesquiterpene, has demonstrated antimicrobial effects; however, its biological responses in Gram-negative bacteria and associated membrane interactions remain insufficiently characterized. In this study, the growth inhibitory effect of BCP against *E. coli* ATCC 25922 was evaluated through OD595 growth kinetics, while hemocompatibility was assessed using sheep erythrocytes, and cannabidiol (CBD) was included as a comparative control. To investigate membrane-associated effects, differential scanning calorimetry (DSC) was performed using DPPE/DPPG (8:2) bilayers as a simplified phospholipid membrane model. BCP inhibited bacterial growth with an IC_50_ of 0.83 mg/mL and exhibited low hemolytic activity (2.92% at 1 mg/mL). DSC analyses revealed concentration-dependent shifts in phase transition temperature and reductions in transition enthalpy (ΔH kJ/mol) 36% and 82% for BCP-5 and CBD-10 according to the control, indicating alterations in lipid organization and membrane thermotropic behavior. In contrast, CBD showed greater growth inhibitory potency (IC_50_ of 0.042 mg/mL) but more pronounced disruption of membrane organization. Overall, these findings suggest that BCP exhibits moderate growth inhibition associated with membrane related effects and low hemolytic activity, providing insights into the relationship between physicochemical properties, membrane interactions, and biological responses.

## 1. Introduction

The emergence and rapid spread of antibiotic-resistant bacterial strains pose a significant threat to public health. The World Health Organization (WHO) has identified antimicrobial resistance as a critical threat to modern healthcare systems due to its impact on therapeutic efficacy, increased infection-related mortality, and rising hospital costs [1,2,3]. The extensive and inappropriate use of antibiotics in human and veterinary medicine, as well as in agriculture, has favored the selection of multidrug-resistant strains that can evade multiple pharmacological mechanisms of action. This situation has driven the search for new therapeutic strategies and the development of antimicrobial agents based on naturally occurring compounds, which can exert their activity through unconventional mechanisms, including interaction with bacterial membranes, alteration of permeability and destabilization of lipid organization [2,4,5].

Plant secondary metabolites have been considered promising alternatives due to their diverse biological properties, including antioxidant, anti-inflammatory, acaricide effect, and antimicrobial activities [1,6]. These compounds include alkaloids, flavonoids, phenolic compounds, terpenes, and lipophilic compounds derived from essential oils. The latter two groups have demonstrated a remarkable capacity to interact with biological membranes, altering lipid organization, and modifying cell permeability [4,7,8]. Such effects can compromise the structural integrity of the membrane and affect essential processes such as solute transport and maintenance of membrane potential in pathogenic microorganisms [7]. Although these mechanisms have been extensively described, their effectiveness in Gram-negative bacteria can be reduced by the presence of an outer membrane rich in lipopolysaccharides, which acts as a selective barrier against hydrophobic compounds [5]. Even so, the ability of certain terpenes to interact with membrane systems and alter their lipid organization makes them candidates for development of antimicrobial strategies.

β-Caryophyllene (BCP) has received increasing attention due to its distinctive physicochemical and pharmacological properties. It is a bicyclic sesquiterpene hydrocarbon (C_15_H_24_) with cannabimimetic activity present in the essential oils of aromatic plants such as *Piper nigrum*, *Syzygium aromaticum*, *Origanum vulgare*, and *Cannabis sativa* [9,10]. BCP is composed primarily of single and double carbon–carbon bonds and lacks polar functional groups, which confers a highly hydrophobic character and a strong affinity for lipid environments (Table 1) [11]. These properties favor its partitioning into lipid bilayers and its interaction with hydrophobic regions of biological membranes, potentially influencing the organization of lipid microdomains and membrane stability [4,12,13].

Scientific interest in BCP has increased due to its classification as a functional phytocannabinoid. Unlike many other terpene compounds, BCP acts as a selective agonist of the cannabinoid receptor type 2 (CB2), enabling modulation of immunological and inflammatory processes without producing the psychoactive effects associated with activation of the CB1 receptor [11]. This property has contributed to its classification as a “dietary cannabinoid,” capable of exerting biologically relevant effects through signaling pathways of the peripheral endocannabinoid system [9,11]; furthermore, its highly lipophilic nature, which it shares with other phytocannabinoids such as cannabidiol (CBD), favors its association with lipid environments and supports the hypothesis that receptor-independent interactions with lipid bilayers may also contribute to its biological effects [4,13,14], thereby expanding biomedical interest in this compound.

**Table 1:** Physicochemical and biophysical properties of β-Caryophyllene (BCP).

	BCP	Biophysical	References
Chemical Structure	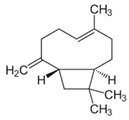	Rigid hydrophobic scaffold favors insertion into lipid bilayers	[10,11]
Chemical Formula	C_15_H_24_	-	[10]
Chemical class a	Bicyclic sesquiterpene hydrocarbon	Hydrophobic structure favor interaction with lipid core	[11]
Molecular Weight	204.36 g/mol	Influences diffusion and partition with the membrane	[9]
Log P	~6.3	Highly lipophilic enables strong partition into lipid membranes	[12]
Key functional groups	Hydrocarbon skeleton with double bonds (no polar functional groups)	Hydrogen bonding capacity absent, favor localization in hydrophobic membrane regions	[11,13,15,16]
Membrane affinity	High affinity for lipid bilayers and hydrophobic membrane regions	Promotes insertion into membrane and interaction with phospholipid acyl chains	[13,16]

Several studies have documented multiple biological activities associated with BCP, including anti-inflammatory, antioxidant, analgesic, and antimicrobial effects [9]. Antibacterial activity has also been observed against different Gram-positive microorganisms suggesting alterations in cell surface potential, increased membrane permeability, and the release of intracellular material [17], as well as partially modulating bacterial efflux systems [18]. These observations support the hypothesis that membrane-associated interactions involving changes in lipid packing and bilayer organization may contribute to the antibacterial activity of BCP [5,8,19]. Although most experimental evidence reports its effects against Gram-positive bacteria, some studies have explored the activity of β-caryophyllene against Gram-negative bacteria. Selestino Neta et al. (2017) [20] reported antibacterial activity against *Escherichia coli* ATCC 8739, with a MIC of approximately 1.0 mg/mL and a progressive reduction in bacterial viability during cell death kinetics studies. These findings suggest that BCP may exert antibacterial effects in Gram-negative systems, although the mechanisms involved under these conditions have not yet been fully elucidated.

Techniques such as DSC enable the analysis of thermotropic changes in membrane phospholipids following exposure to antimicrobial compounds, providing insights into alterations in fluidity, lipid organization, and bilayer stability [4]. Biophysical studies have demonstrated that lipophilic compounds such as BCP can be incorporated into phospholipid bilayers and modify their thermal behavior, suggesting a potential role of membrane-associated effects in their biological activity [12,13]. This type of analysis provides complementary information to biological assays by helping to characterize the interactions of lipophilic compounds with simplified membrane models and their possible relationship with observed cellular responses.

The present study aims to evaluate the growth inhibitory, effect of BCP against *Escherichia coli* ATCC 25922, its hemolytic activity in ovine erythrocytes, and its interaction with DPPE/DPPG (80:20, mol/mol) bilayers used as a simplified model of the major phospholipid environment of the *E. coli* inner membrane. CBD was included as a reference compound. The study integrates bacterial growth kinetics, hemolytic evaluation, and DSC-based biophysical characterization to investigate changes in lipid bilayer organization and thermotropic behavior.

## 2. Materials and Methods

### 2.1. Bacterial Growth Kinetics in the Presence of β-Caryophyllene

The microorganism used in this study was *E. coli* ATCC 25922, obtained from the American Type Culture Collection (ATCC). This strain is recommended by the Clinical & Laboratory Standards Institute (CLSI) as a quality control strain for antimicrobial susceptibility testing. A fresh bacterial culture was used to prepare the inoculum, which was adjusted to a 0.5 McFarland standard using Difco™ Nutrient Broth [Becton, Dickinson and Company (BD), Franklin Lakes, NJ, USA] (OD_595nm_ = 0.125). Growth kinetics were subsequently evaluated in nutrient broth using a 96-well microplate photometer [21] (Multiskan™ FC, Thermo Fisher Scientific Inc., Singapore) at 37 ± 2 °C under continuous internal shaking. Each well contained a final volume of 200 µL, including 100 µL of bacterial suspension adjusted to 1.5 × 10^7^ CFU/mL. BCP and CBD, used as control, were tested at final concentrations of 0.031, 0.0625, 0.125, 0.25, 0.5, and 1.00 mg/mL. Stock solutions of both compounds were prepared in DMSO and subsequently diluted in nutrient broth to reach the desired concentrations. The final DMSO concentration in the assay ranged from 1.22% to 0.037% (*v*/*v*), decreasing proportionally with compound dilution. Bacterial growth and substrate assimilation were monitored by measuring the optical density (OD) at 595 nm every hour for 24 h. Time zero corresponded to freshly inoculated axenic cultures collected during the exponential growth phase. Specific growth rates (*μ*) were estimated by plotting the natural logarithm of OD versus time. All experiments were conducted in duplicate independent.

### 2.2. Preparation of Vesicles as a Simplified Model of E. coli-like Membranes

Lombardi et al. (2017) [22] used liposomes composed of the unsaturated phospholipids 1,2-dioleoyl-sn-glycero-3-phosphoethanolamine (DOPE), 1,2-dioleoyl-sn-glycero-3-phospho-(1-rac-glycerol) sodium salt (DOPG) at an 80:20 molar ratio as a simplified model of the major phospholipid environment of the *E. coli* inner membrane. For DSC experiments, the corresponding saturated phospholipids, specifically 1,2-dipalmitoyl-sn-glycero-3-phosphoethanolamine (DPPE) and 1,2-dipalmitoyl-sn-glycero-3-[phospho-rac-(1-glycerol)] (DPPG), provide a well-defined phase transition within an experimental accessible temperature range, as previously described. Based on this approach, a DPPE/DPPG mixture (80:20 molar ratio) was used in the present study as a simplified inner-membrane phospholipid model. The phospholipids were purchased from Avanti Polar Lipids (Alabaster, AL, USA).

Multilamellar vesicles (MLVs) were prepared at a final total lipid concentration of 0.5 mM. The lipids were dissolved in methanol/chloroform (70/30 *v*/*v*) in an amber scintillation vial. A thin lipid film was obtained by evaporating the organic solvent under a nitrogen stream, then using a degassing station (TA Instruments, Newcastle, DE, USA) at 55 °C for 30 min, stirring at 130 rpm to remove all residual organic solvents. Subsequently, the film was hydrated with Milli-Q water at 60 °C and finally stirred at 230 rpm for 2 h to obtain a multilamellar vesicle (MLV) suspension.

### 2.3. Differential Scanning Calorimetry

Measurements were performed using Nano DSC and TA Instruments (New Castle, DE, USA). For all experiments, MLVs were used. A volume of 300 μL of lipid mixtures (0.5 mM) in the absence or presence of BCP or CBD at 10 and 5 mol% was placed in the calorimetry vessel. Successive heating cycles were performed for each sample, operating at a rate of 1 °C min^−1^ in the temperature range of 25 to 80 °C, after a 600 s equilibration time. Thermograms were analyzed to determine the main phase transition temperature (Tm), enthalpy (ΔH). All experiments were performed in triplicate.

### 2.4. Hemolytic Activity Assay of Test Compounds Using Ovine Erythrocytes

Whole defibrinated sterile sheep blood free of antibiotics and additives (DIBICO SC50FC-111225B, Cuautitlán Izcalli, Estado de México, México) was used for the isolation of red blood cells (RBCs). A 1 mL aliquot was centrifuged at 4000 rpm for 5 min at 4 °C. The supernatant was discarded, and the resulting pellet was resuspended in 4 mL of sterile phosphate-buffered saline (PBS, pH 7.0). This washing step was repeated three times or until the supernatant appeared completely clear indicating the effective removal of plasma and soluble components. After the final wash, the erythrocyte pellet was diluted with 99 mL of sterile physiological saline solution 0.9% sodium chloride (NaCl), pH 7.0 to obtain a 1% *v*/*v* RBC suspension.

For the hemolytic assay, a stock solution (8 mg/mL in DMSO) was serially diluted in physiological saline to obtain final concentrations of 1.0, 0.5, 0.25, 0.125, 0.0625, 0.031, 0.015, 0.007, 0.0038 and 0.0019 mg/mL. The corresponding final DMSO concentration ranged from 1.22% to 0.0023% (*v*/*v*), decreasing proportionally with compound dilution. A saline-only sample was used as a negative control.

Next, 200 μL of the 1% RBC suspension was added to each tube. The tubes were gently mixed and incubated in a water bath at 37 °C for 60 min. After incubation, the samples were centrifuged at 4000 rpm for 5 min, and 300 μL of each supernatant were transferred to BioSystems cuvettes. Absorbance was measured at 405 nm using a Y15 automated spectrophotometer (BioSystems, Zapopán, Jalisco, México), with each concentration tested in triplicate. The percentage of hemolysis was calculated using the following equation [23,24]:

$$\%\;\mathrm{Hemolysis}=(\frac{{OD}_{test}-{OD}_{neg}}{{OD}_{pos}-{OD}_{neg}})*100$$

where *OD_test_* is the optical density of the treated sample, *OD_ne__g_* is the negative control (untreated erythrocytes), and *OD_pos_* is the positive control (100% lysis using 10% *w*/*v* Tween-20). This method enables quantification of the hemolytic potential of test compounds on erythrocyte membranes.

### 2.5. Statistical Analysis

The effects of compound type (BCP and CBD) and concentration (1.0, 0.5, 0.25, 0.125, 0.0625, 0.0312 mg/mL, and 0 mg/mL as control) on the specific growth rate (*μ*) of *Escherichia coli* were evaluated using a multifactorial categorical design comprising 28 experimental runs, including duplicate measurements for each treatment. The resulting data were analyzed by analysis of variance (ANOVA) followed by Fisher’s least significant difference (LSD) test for mean comparison, with statistical significance established at a 95% confidence level (*p* < 0.05). Probit regression analysis was performed to evaluate the relationship between compound concentration and the observed mortality percentage for both BCP and CBD, allowing the estimation of the median inhibitory concentration (IC_50_) and other lethality parameters (IC_90_). All statistical analyses were analyzed using Statgraphics Centurion XVI Version 16.2.04 software (Virginia, VA, USA).

Differential scanning calorimetry (DSC) data were analyzed using NanoAnalyze software (TA Instruments, New Castle, DE, USA), and graphical representations were generated using GraphPad Prism version 10.3.1 (GraphPad Software, Boston, MA, USA).

## 3. Results

### 3.1. Bacterial Growth of Escherichia coli and Determination of IC_50_ and IC_90_

**Figure 1:**
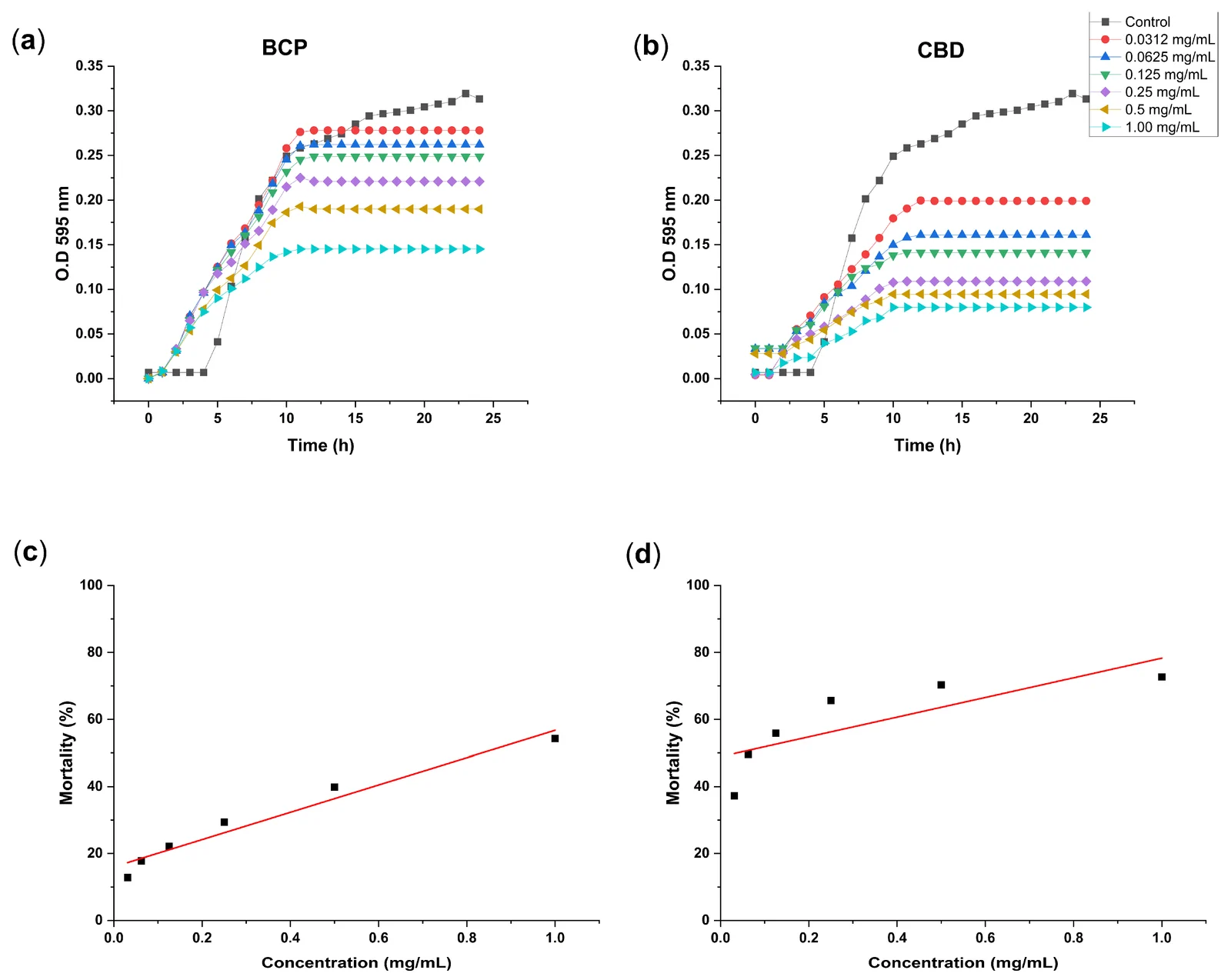
Growth curves of *E. coli* ATCC 25922 and Probit regression analysis of BCP (**a**,**c**) and CBD (**b**,**d**) treatments. IC_50_ and IC_90_ values were estimated from the fitted regression models.

The growth inhibitory effect of BCP against *E. coli* ATCC 25922 was evaluated through bacterial growth kinetics analysis based on OD_595_ measurements (Figure 1a). The control group exhibited a characteristic sigmoidal growth pattern, reaching an optical density (OD_595_ nm) of approximately 0.31 after 24 h. BCP treatment resulted in a concentration-dependent reduction in bacterial growth. Low concentrations (0.0312 and 0.0625 mg/mL) produced only minor changes compared with the control, whereas concentrations ≥0.25 mg/mL markedly reduced bacterial growth throughout the incubation period. Based on Probit regression of the OD595 growth inhibition data, the model estimated IC_50_ and IC_90_ values of 0.83 mg/mL and 1.95 mg/mL, respectively. Because the highest experimental concentration evaluated was 1.0 mg/mL, the IC_90_ represents an extrapolated estimate of the fitted regression model rather than an experimentally observed value.

CBD was evaluated as a reference phytocannabinoid (Figure 1b) and exhibited a stronger growth inhibitory effect at lower concentrations, with an estimated IC_50_ value of 0.042 mg/mL (95% CI: 0.0418–0.0420 mg/mL). Compared with BCP, CBD required a lower concentration to achieve 50% inhibition of bacterial growth based on OD_595_ measurements. Probit regression estimated an IC_90_ value of 1.66 mg/mL (95% CI: 1.6578–1.6585 mg/mL). Because the highest concentration experimentally evaluated was 1.0 mg/mL, this IC_90_ represents an extrapolated estimate of the fitted model rather than an experimentally observed value. Overall, both BCP and CBD exhibited concentration-dependent growth inhibitory effects against *E. coli* ATCC 25922 under the experimental conditions evaluated.

### 3.2. Effect of Compound Type CBD and BCP and Concentration on the Specific Growth Rate (μ) of Escherichia coli

The specific growth rate (*μ*) of *E. coli* was influenced by compound type and concentration (Table 2). Relative to the control (*μ* ≈ 0.29 h^−1^), exposure to BCP and CBD reduced bacterial growth kinetics across the evaluated concentration. Increasing concentrations were associated with progressively lower *μ* values for both compounds, indicating concentration-dependent inhibition of bacterial proliferation.

**Figure 2:**
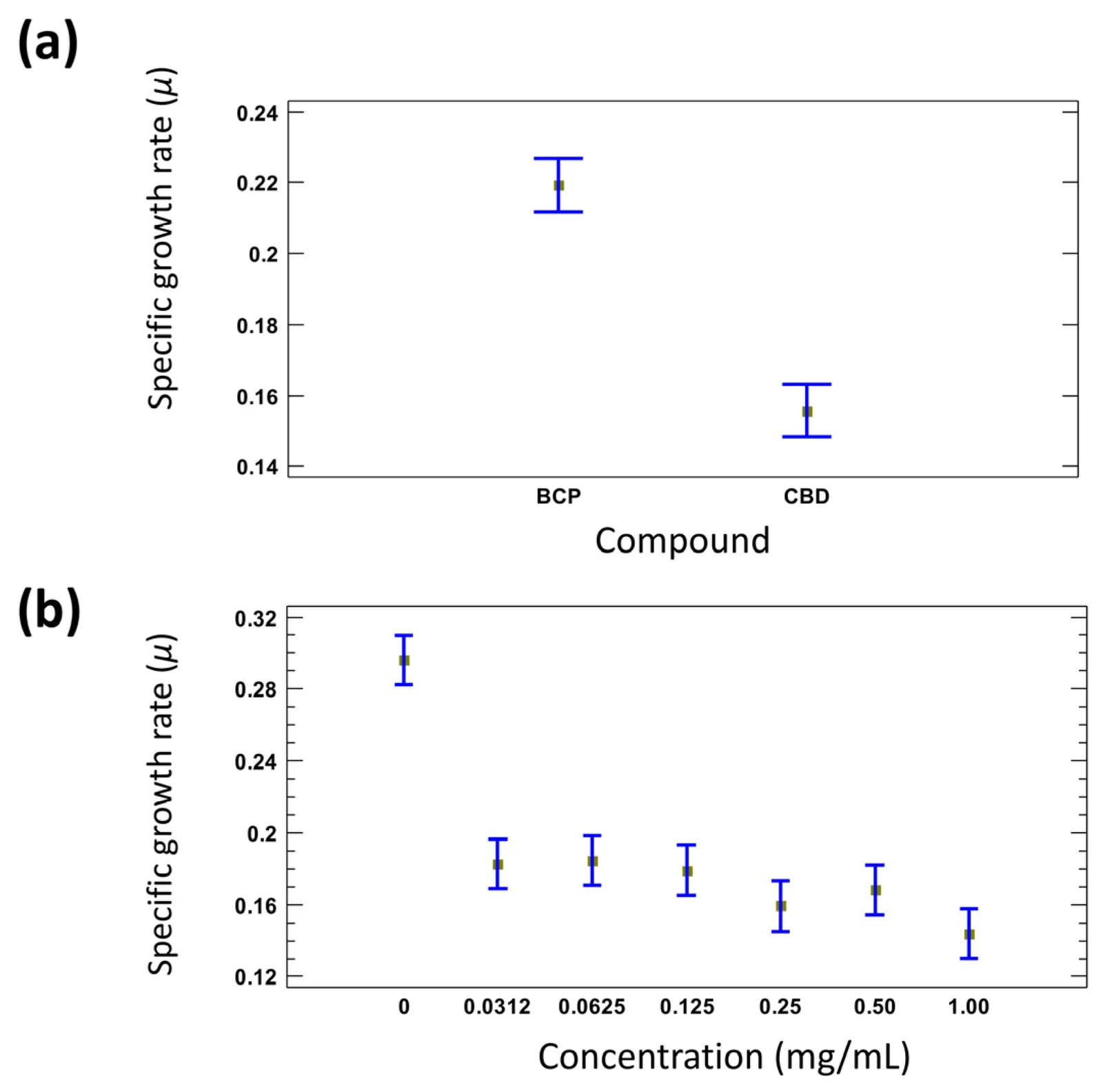
Effect of BCP and CBD (**a**) and concentrations (**b**) on the specific growth rate (*μ*) of *E. coli*. Data are presented as mean ± standard deviation of duplicate experiments (n = 2), (*p* < 0.05).

BCP-treated cultures exhibited *μ* values ranging from 0.1665 ± 0.005 to 0.2278 ± 0.007 h^−1^, whereas CBD treatment produced lower growth rates, ranging from 0.1179 ± 0.006 to 0.1445 ± 0.029 h^−1^. These results demonstrate that CBD exerted a stronger reduction in bacterial growth kinetics than BCP under the evaluated conditions. The effects of compound type, concentration, and their interaction on *μ* were analyzed using a multifactorial categorical design (Table 3, Figure 2). Analysis of variance (ANOVA) revealed that both factors significantly affected the specific growth rate of *E. coli* (*p* < 0.05). In addition, the interaction between compound type and concentration was statistically significant (*p* = 0.0495), indicating that the effect of concentration on bacterial growth kinetics differed depending on the compound evaluated. Figure 2a shows the distribution of mean *μ* values grouped according to compound type, highlighting distinct growth-inhibitory profiles between BCP and CBD. Similarly, Figure 2b presents the mean *μ* values according to concentration. The untreated control exhibited the highest specific growth rate, whereas all evaluated compound concentrations produced lower *μ* values. However, the magnitude of the reduction did not follow a strictly progressive pattern across the concentration range.

### 3.3. Hemolytic Activity

The hemolytic activity of BCP and CBD was evaluated using ovine erythrocytes as a model for membrane interaction and preliminary membrane toxicity. BCP exhibited negligible hemolytic activity across the evaluated concentration range (0.0019–1 mg/mL). No measurable hemolysis was detected at concentrations below 1 mg/mL, while the highest concentration tested (1 mg/mL) resulted in only 2.92 ± 0.44% hemolysis.

**Figure 3:**
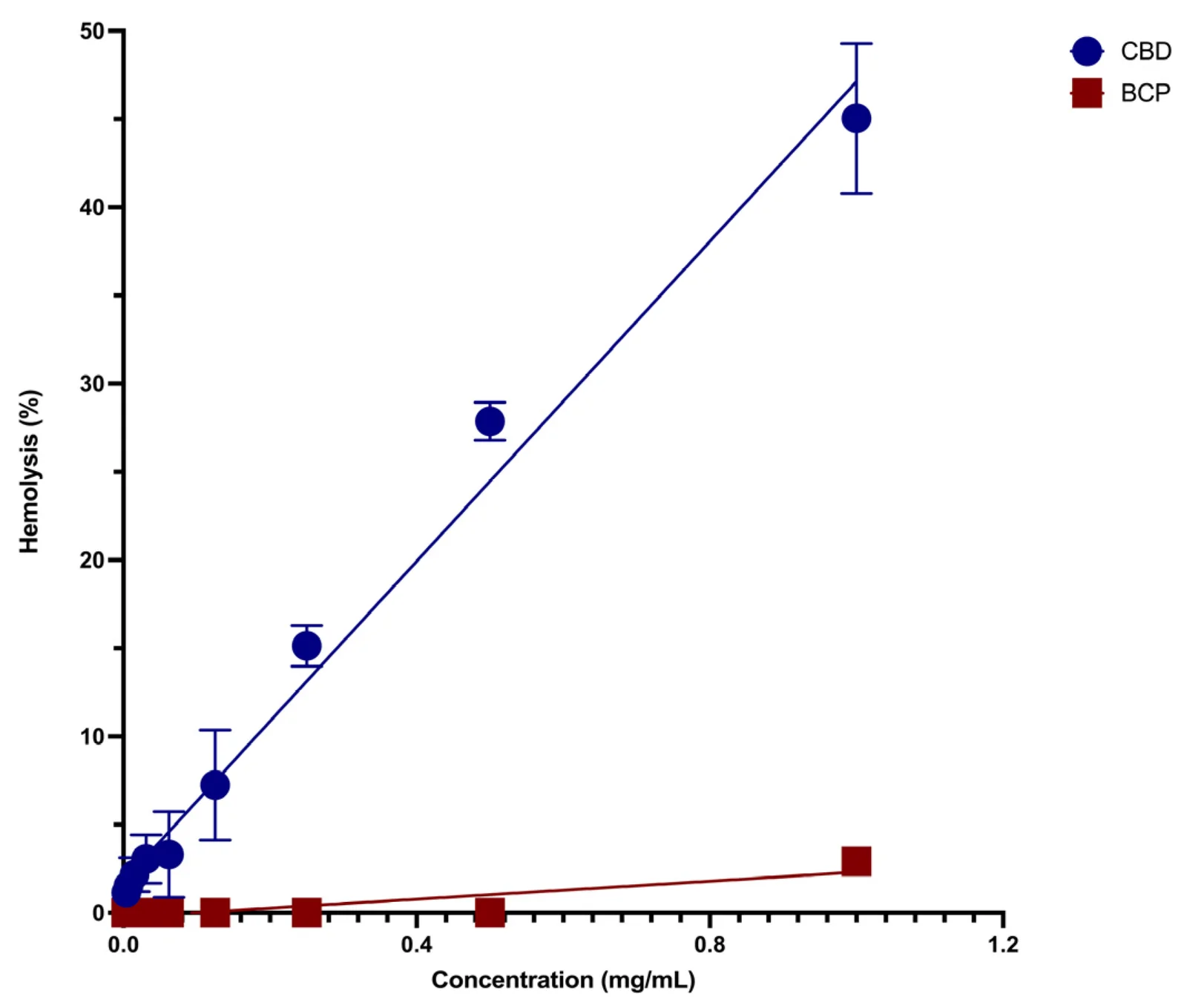
Hemolytic activity of cannabidiol (CBD) and β-caryophyllene (BCP) against red blood cells at increasing concentrations (0–1 mg/mL). The data represent the mean ± standard deviation (n = 3). The line shows the fitted linear regression model describing the concentration-dependent hemolytic response.

CBD showed a concentration-dependent increase in hemolytic activity over the range of 0.0019 to 1 mg/mL (Figure 3). Hemolysis remained below 5% at concentrations between 0.0019 and 0.062 mg/mL. A pronounced increase was observed at concentrations ≥ 0.125 mg/mL, reaching 27.86 ± 1.07% at 0.5 mg/mL and 45.03 ± 4.45% at 1 mg/mL.

The fitted regression model showed a positive linear relationship between CBD concentration and hemolytic activity. Overall, the evaluated compounds exhibited distinct erythrocyte interaction profiles, with CBD producing substantially higher hemolytic effects than BCP under the tested conditions.

### 3.4. Assessment of Bacterial Membrane Integrity

**Figure 4:**
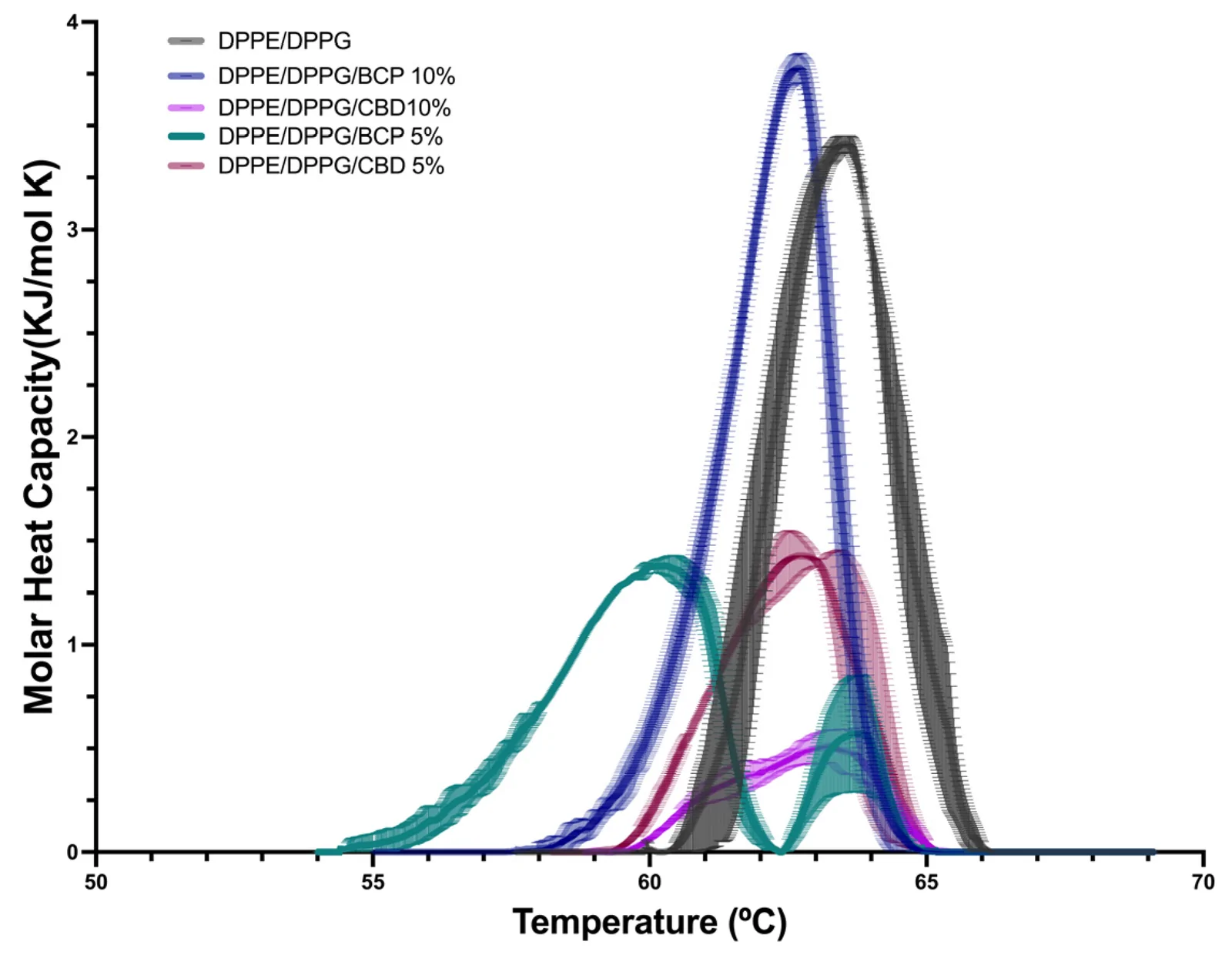
Differential scanning calorimetry (DSC) thermograms of synthetic DPPE/DPPG membranes in the absence (control) and presence of β-caryophyllene (5 and 10%) and cannabidiol (5 and 10%).

The thermotropic behavior of the synthetic DPPE/DPPG membrane in the absence and presence of BCP and CBD was evaluated using differential scanning calorimetry (DSC) (Figure 4). The control membrane exhibited a well-defined main transition in the 62–64 °C range, with a transition temperature (Tm) of 63.55 ± 0.08 °C and a transition enthalpy (ΔH) of 8.93 ± 0.08 kJ/mol (Table 4). The thermogram displayed a narrow and symmetrical peak, characteristic of a cooperative gel-to-liquid crystalline phase transition in saturated phospholipid bilayers.

BCP modified the thermotropic profile in a concentration-dependent manner. At 5%, the incorporation of BCP reduced Tm to 60.20 ± 0.20 °C and decreased ΔH to 5.70 ± 0.03 kJ/mol, accompanied by transition broadening relative to the control membrane. At 10%, the membrane system exhibited a Tm of 62.70 ± 0.12 °C and an increased ΔH of 9.12 ± 0.05 kJ/mol. Under this condition, the thermogram displayed a broader transition profile with greater peak intensity than the control membrane.

These changes indicate that BCP altered the thermotropic behavior of the membrane depending on concentration. The reduction in Tm and ΔH observed at 5% was associated with decreased transition cooperativity, whereas the increase in ΔH observed at 10% reflected a distinct transition profile relative to the lower concentration treatment. The broadening of the thermograms under both conditions indicates changes in membrane organization and phase transition behavior. Considering the hydrophobic nature of BCP and its affinity for lipid bilayers [9,13], these effects are consistent with interactions between BCP and the hydrophobic region of the membrane.

CBD also modified the thermotropic profile of DPPE/DPPG membranes. At 5%, ΔH decreased to 4.27 ± 0.05 kJ/mol with a Tm of 62.79 ± 0.25 °C, whereas at 10%, ΔH decreased markedly to 1.65 ± 0.01 kJ/mol while Tm remained at 63.34 ± 0.20 °C. The corresponding thermograms exhibited broader and less defined transitions than those observed for the control and BCP-treated membranes.

Overall, both phytocannabinoids altered the thermotropic behavior of DPPE/DPPG membranes, producing distinct effects on transition enthalpy, transition temperature, and phase transition profiles.

## 4. Discussion

The growth inhibitory effect observed for β-caryophyllene (BCP) against *Escherichia coli* ATCC 25922 is relevant in the broader context of the importance of *E. coli* in food safety and public health, particularly serotype O157:H7, because associated as it causes diseases ranging from mild gastroenteritis to severe conditions that can lead to death due to the effect of its potent bacterial toxin [25,26,27]. The increasing prevalence of antimicrobial resistance has intensified the search for alternative antimicrobial agents, including bioactive compounds derived from natural products [28]. Natural products and their derivatives represent an important source of structurally diverse antibacterial compounds with potential therapeutic applications. Among these, phytocannabinoids and lipophilic terpenoids have growing attention due to their antimicrobial activity and membrane-associated biological effects [29,30,31].

BCP is a bicyclic sesquiterpene widely distributed in aromatic and medicinal plants, including black pepper, oregano, cinnamon, basil, clove, and *Cannabis sativa* [10,32,33,34]. Its biological relevance has been associated with its high hydrophobicity and affinity for lipid environments, which may facilitate interactions with biological membranes [9,13]. Previous studies using model membranes and calorimetric approaches have shown that BCP and other lipophilic terpenoids can modify lipid organization and thermotropic properties, supporting the relevance of membrane-associated effects in their biological activity [13,35]. In addition to its membrane-related properties, BCP has attracted interest due to its antioxidant, anti-inflammatory, and antimicrobial activities [9,17,18].

Based on these characteristics, the present study evaluated growth inhibitory effect of BCP against *Escherichia coli* ATCC 25922 through bacterial growth kinetics analysis, erythrocyte hemolysis assays as a membrane interaction model, and differential scanning calorimetry (DSC) to characterize its effects on model membrane thermotropic behavior. Cannabidiol (CBD), a phytocannabinoid with reported membrane-active antimicrobial properties, was included as a reference compound for comparative analysis [14,29,30].

The present findings show that BCP inhibited *E. coli* growth and modified the thermotropic profile of the synthetic membrane model for both phytocannabinoids. BCP exhibited a moderate growth inhibitory effect against *E. coli* based on OD_595_ measurements (IC_50_ = 0.8317 mg/mL). Selestino Neta et al. (2017) reported an MIC of approximately 1.0 mg/mL for BCP against *E. coli* ATCC 8739 [20]. Because MIC and OD_595_-derived IC_50_ values represent different experimental endpoints, they cannot be directly compared. Nevertheless, both studies report inhibitory effects within a similar nominal concentration range. Differences in bacterial strain, experimental conditions, and endpoint determination may contribute to variations in the observed responses.

In comparison, CBD exhibited a stronger growth inhibitory effect against *E. coli* based on OD_595_ measurements (IC_50_ = 0.0419 mg/mL), although this effect was accompanied by markedly higher hemolytic activity, reaching approximately 45% at 1 mg/mL. The pronounced hemolytic activity of CBD is consistent with its high lipophilicity and amphiphilic character, which favor interactions with lipid bilayers. Consistent with this behavior, CBD and Δ^9^-tetrahydrocannabinol (THC) have been shown to alter erythrocyte membrane integrity, rheological properties, and red blood cell morphology at elevated concentrations [36].

In comparison, BCP exhibited minimal hemolytic activity (≈2.9% at 1 mg/mL), suggesting lower membrane toxicity toward cholesterol-rich erythrocyte membranes while maintaining antibacterial activity against *E. coli*. This differential behavior indicates distinct membrane interaction profiles between CBD and BCP and supports the hypothesis that membrane-associated antibacterial activity can occur through structurally different modes of lipid perturbation. Similar selective membrane-associated effects have been described for lipophilic terpenoids capable of partitioning into phospholipid bilayers and modulating membrane physicochemical properties without inducing extensive membrane disruption [9,13,35].

Differential scanning calorimetry performed using DPPE/DPPG (8:2) bilayers, selected as a model membrane system resembling the phospholipid composition of *Escherichia coli*, showed that both phytocannabinoids altered membrane thermotropic behavior, although with markedly different profiles. CBD produced substantial reductions in transition enthalpy accompanied by extensive peak broadening and loss of transition cooperativity, indicating pronounced disruption of lipid organization within the bilayer. These alterations are consistent with interfacial insertion of CBD near phospholipid headgroup regions, promoting packing defects, membrane heterogeneity, and increased disorder within the membrane structure [37]. Similar membrane-associated effects of phytocannabinoids have been described previously in artificial lipid bilayers and bacterial membranes, where alterations in lipid packing and membrane organization were associated with increased membrane permeability and destabilization of membrane-dependent physiological processes [29,35].

**Figure 5:**
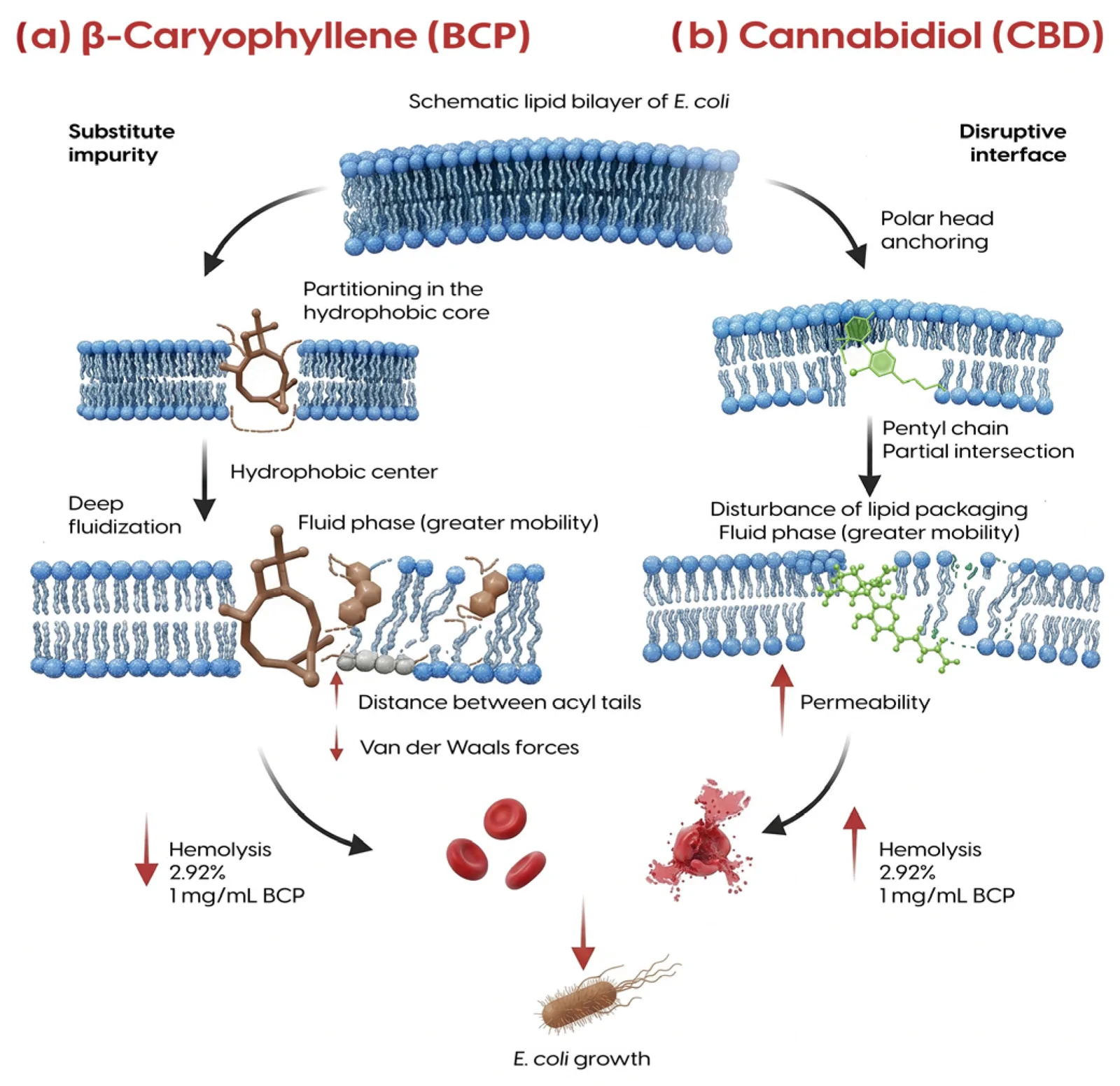
Proposed schematic of the mechanism of action of β-caryophyllene (BCP) and cannabidiol (CBD) on the cell membrane of *E. coli*. (**a**) BCP interaction with the lipid bilayer, showing minimal disruption of membrane integrity; (**b**) CBD-induced membrane destabilization, leading to increased permeability and structural damage.

The pronounced membrane perturbation induced by CBD may contribute to dissipation of the membrane potential (Δψ), an essential component of the proton motive force (PMF), thereby compromising membrane-associated energy transduction processes, as suggested by previous studies investigating the antibacterial mechanism of cannabidiol [29,38]. In addition, CBD has been reported to interfere with membrane-associated adaptive responses in Gram-negative bacteria, including reduced outer membrane vesicle production, thereby increasing bacterial susceptibility to environmental stress and antibacterial agents [29,39]. The elevated hemolytic activity observed for CBD in the present study further supports its strong interaction with lipid bilayers and reduced selectivity toward eukaryotic membranes. The membrane-associated effects proposed from the microbiological, hemolytic, and calorimetric analyses are summarized schematically in Figure 5.

Collectively, these observations support the hypothesis that CBD acts primarily as an interfacial membrane destabilizer capable of compromising both the structural organization and functional integrity of bacterial membranes. The pronounced decrease in transition enthalpy, extensive peak broadening, and marked loss of cooperativity observed in the DSC analyses are consistent with substantial perturbation of lipid packing and membrane organization. Similar membrane perturbations have been described for membrane-active compounds interacting with artificial phospholipid bilayers, where membrane insertion promotes lipid disorder, increased permeability, and destabilization of membrane-associated processes [37]. Such membrane perturbations are expected to compromise membrane-associated energy transduction processes, including maintenance of the proton motive force, ultimately reducing bacterial viability. The elevated hemolytic activity observed for CBD further supports its strong interaction with lipid bilayers and reduced selectivity toward cholesterol-rich eukaryotic membranes.

BCP exhibited a markedly different thermotropic and biological profile. As a highly hydrophobic bicyclic sesquiterpene lacking polar functional groups, BCP is expected to partition preferentially within the hydrophobic core of phospholipid bilayers rather than at the membrane interface [9,13]. BCP has been shown to incorporate into phospholipid bilayers, where it modulates membrane thermotropic behavior through hydrophobic interactions within the lipid phase [13]. At lower concentrations, the reduction in transition enthalpy observed in the present study suggests decreased phospholipid cooperativity and increased acyl chain mobility, consistent with membrane fluidization induced by the presence of hydrophobic molecules acting as substitutional impurities within the bilayer [40,41]. Unlike CBD, these alterations were not associated with extensive membrane disruption or high hemolytic activity, suggesting a more selective membrane interaction profile.

Interestingly, the thermodynamic behavior observed at 10% BCP suggests a concentration-dependent reorganization of the membrane system. Although the thermograms remained broader than the control profile, the increase in ΔH relative to the 5% treatment suggests the emergence of more energetically stable lipid interactions within the bilayer. Similar alterations in thermotropic behavior have been associated with membrane reorganization and heterogeneous lipid packing in studies of membrane-active compounds, including β-caryophyllene and antimicrobial peptides [13,40]. Under these conditions, BCP may promote heterogeneous membrane organization, potentially characterized by coexisting fluidized and more ordered lipid domains, rather than generalized membrane destabilization. This interpretation is further supported by the low hemolytic activity despite measurable antibacterial effects.

The membrane interactions suggested by the DSC results may contribute to alterations in membrane-dependent bacterial processes. Perturbations in bilayer organization and lipid dynamics have been associated with changes in membrane permeability, membrane-associated energy transduction, and the functionality of transmembrane transport systems [13,37]. However, these effects were not directly evaluated in the present study and should be considered as potential mechanisms requiring further investigation. Potential modulation of efflux systems such as AcrAB-TolC could favor intracellular accumulation of toxic compounds and increase bacterial susceptibility to environmental stressors and antimicrobial agents [42,43]. Concurrently, bacterial adaptation to membrane fluidization frequently involves remodeling of lipid composition to restore membrane rigidity and physicochemical stability, an energetically demanding process associated with altered cellular metabolism and reduced bacterial fitness [44,45].

Taken together, the microbiological, hemolytic, and calorimetric findings support a differential membrane-associated model for CBD and BCP, summarized schematically in Figure 5. CBD may interact with the interfacial region of membrane destabilizer associated with strong disruption of lipid organization and reduced membrane selectivity, whereas BCP may behave as a hydrophobic core modulator capable of altering membrane dynamics while maintaining comparatively low hemolytic effects. This differential membrane interaction profile may contribute to the observed differences between growth inhibition and hemolytic effects observed for both compounds.

The membrane-associated effects observed for CBD are consistent with its structural characteristics as a lipophilic terpenophenolic phytocannabinoid containing aromatic and hydroxylated moieties capable of interacting with both hydrophobic and interfacial regions of lipid bilayers. These physicochemical properties favor membrane insertion, alteration of lipid packing, and modulation of membrane organization [29,37]. Previous studies have demonstrated that CBD exhibits antibacterial activity against a broad range of microorganisms, particularly Gram-positive bacteria, while activity against Gram-negative species appears to be more strongly influenced by membrane permeability barriers and experimental conditions [29,30]. The lower susceptibility frequently reported for Gram-negative bacteria has been associated with the presence of an outer membrane enriched in lipopolysaccharides, which restricts penetration of hydrophobic compounds and contributes to intrinsic resistance mechanisms [29]. Nevertheless, the antibacterial activity observed against *E. coli* in the present study suggests that both CBD and BCP may interact with bacterial envelope components in a manner that contributes to growth inhibition under the evaluated conditions.

The comparatively lower hemolytic activity and distinct calorimetric behavior observed for BCP suggest a membrane interaction mechanism fundamentally different from that of CBD. Due to its highly hydrophobic sesquiterpene structure and lack of polar functional groups, BCP may interact preferentially with the hydrophobic core of the bilayer model, potentially promoting alterations in lipid dynamics without inducing the extensive membrane destabilization associated with CBD [9,13]. Similar membrane-associated effects have been described for membrane-active compounds, where hydrophobic interactions with phospholipid acyl chains modulate membrane physicochemical properties [40,41]. In addition, previous studies have suggested that the antimicrobial activity of essential oils rich in BCP may involve synergistic interactions among multiple terpenoid constituents, enhancing membrane perturbation and antibacterial efficacy [46].

Beyond its antimicrobial activity, BCP has attracted growing interest due to its diverse biological properties, including anti-inflammatory, antioxidant, analgesic, immunomodulatory, and antiparasitic activities [9]. The combination of membrane-associated antibacterial activity, low hemolytic potential, and broad biological compatibility highlights BCP as a promising bioactive terpenoid for future studies investigating membrane-associated growth inhibition mechanisms and its potential applications in antimicrobial research.

Taken together, these findings should be interpreted with consideration of certain limitations. A standardized minimum inhibitory concentration (MIC) assay was not performed; therefore, antibacterial activity was assessed by concentration-dependent growth inhibition and IC_50_ determination. In addition, the phospholipid membrane model used for DSC represents a simplified system that does not fully reproduce the structural complexity of the *E. coli* cell envelope. Future studies incorporating standardized susceptibility testing and complementary analyses of membrane permeability, membrane potential, proton motive force, and efflux activity could provide further mechanistic insight into the effects of CBD and BCP.

## 5. Conclusions

This study demonstrates that BCP induces a concentration-dependent inhibitory effect on the growth of *E. coli* ATCC 25922 under the experimental conditions evaluated, which was associated with membrane-related effects characterized by alteration in lipid organization and bilayer cooperativity. Differential scanning calorimetry analyses suggest that BCP interacts with the hydrophobic region of the membrane model, promoting changes in membrane dynamics and thermotropic behavior without inducing the extensive membrane perturbation observed for CBD.

Although CBD had a stronger growth inhibitory effect based on OD_595_ measurements, BCP displayed markedly lower hemolytic activity, suggesting a more selective interaction with the evaluated membrane models. The combination of growth inhibition, low hemolytic potential, and membrane-modulating behavior highlights BCP as a promising bioactive terpenoid for further investigation as a membrane-interacting compound. Further studies are necessary to clarify the molecular mechanisms associated with its effects on membrane energetics, permeability, and to evaluate its antibacterial potential through standardized susceptibility assays (MIC/MBC) and viability-based approaches.

## Figures and Tables

**Table 2:** Experimental Design Matrix on the Specific Growth Rate [*µ* (h^−1^)] of *E. coli* in CBD and BCP.

	Specific Growth Rate (*µ*)	
Concentration (mg/mL)	CBD	BCP
1.00	0.1411 ± 0.014	0.1665 ± 0.005
0.50	0.1293 ± 0.028	0.2063 ± 0.004
0.25	0.1179 ± 0.006	0.2000 ± 0.010
0.125	0.1404 ± 0.002	0.2174 ± 0.001
0.0625	0.1408 ± 0.005	0.2278 ± 0.007
0.0312	0.1445 ± 0.029	0.2206 ± 0.015
0.00	0.29 ± 0.033	0.29 ± 0.033

**Table 3:** ANOVA results of the Multifactor Categoric Design for *µ* ^a^.

Source	Sum of Square	Degree of Freedom	Mean Square	*F*-Value	*p*-Value
Main effects					
A: Compound	0.0283211	1	0.0283211	84.20	0.0000
B: Concentration	0.060081	6	0.0100135	29.77	0.0000
Interactions					
AB	0.00576486	6	0.00096081	2.86	0.0495
Residual	0.00470919	14	0.00033637		
Total (Corrected)	0.0988761	27			

^a^ All *F* ratios are based on the residual mean square error.

**Table 4:** Thermodynamic parameters obtained from DSC profiles of DPPE/DPPG in the absence and presence of BCP and CBD.

Membrane System Composition	ΔH (kJ/mol)	Tm (°C)
DPPE/DPPG	8.93 ± 0.08	63.55 ± 0.08
DPPE/DPPG/BCP 5%	5.70 ± 0.03	60.20 ± 0.20
DPPE/DPPG/BCP 10%	9.12 ± 0.05	62.70 ± 0.12
DPPE/DPPG/CBD 5%	4.27 ± 0.05	62.79 ± 0.25
DPPE/DPPG/CBD10%	1.65 ± 0.01	63.34 ± 0.20

Data are presented as mean ± SD; (n = 3).

## Data Availability

The raw data supporting the conclusions of this article will be made available by the authors on request.

## Abbreviations

The following abbreviations are used in this manuscript:

BCP	β-Caryophyllene
*E. coli*	*Escherichia coli*
ATCC	American Type Culture Collection
EOs	Essential oils
DSC	Differential scanning calorimetry
WHO	The World Health Organization
CBD	Cannabidiol
CB1	Cannabinoid receptor type 1
CB2	Cannabinoid receptor type 2
MIC	Minimal Inhibitory Concentration
CLSI	The Clinical & Laboratory Standards Institute
OD	Optical Density
nm	Nanometer
°C	Grade Celsius
µL	Microliter
CFU	Colony Forming Unit
DMSO	Dimethyl sulfoxide
DOPE	1,2-dioleoyl-sn-glycero-3-phosphoethanolamine
DPPG	1,2-dipalmitoyl-sn-glycero-3-phosphoethanolamine
MLV	Multilamellar vesicle
mM	Millimolar
h	Hour
rpm	Revolutions per minute
Tm	Transition temperature
ΔH	Enthalpy
PBS	Phosphate-buffered saline
RBC	Red blood cells
NaCl	Sodium chloride
IC_50_	The concentration of a compound required to inhibit 50% of the measured biological response compared with the untreated control.
IC_90_	The concentration of a compound required to inhibit 90% of the measured biological response compared with the untreated control.
*μ*	The specific growth rate
